# Ultrasound-guided gastrocnemius recession: a new ultra–minimally invasive surgical technique

**DOI:** 10.1186/s12891-016-1265-7

**Published:** 2016-10-03

**Authors:** Manuel Villanueva, Álvaro Iborra, Guillermo Rodríguez, Pablo Sanz-Ruiz

**Affiliations:** 1 Avanfi Institute and Unit for Ultrasound-guided Surgery, Hospital Beata María Ana, Calle Orense 32., 28020 Madrid, Spain; 2 Orthopaedic and Trauma Department, Hospital General Universitario Gregorio Marañón, Madrid, Spain

**Keywords:** Ultrasound-guided, Ultra–minimally invasive surgery, Gastrocnemius recession, Achilles tendinopathy, Equinus foot

## Abstract

**Background:**

Isolated gastrocnemius contracture is thought to lead to numerous conditions. Although many techniques have been described for gastrocnemius recession, potential anesthetic, cosmetic, and wound-related complications can lead to patient dissatisfaction. Open and endoscopic recession techniques require epidural anesthesia, lower limb ischemia, and stitches and may lead to damage of the sural nerve, which is not under the complete control of the surgeon at all stages of the procedure.

The purpose of this study was to evaluate the safety and efficacy of a new technique based on ultrasound-guided ultra–minimally invasive gastrocnemius recession.

**Methods:**

We performed a pilot study with 22 cadavers to ensure that the technique was effective and safe. In the second phase, we prospectively performed gastrocnemius recession in 23 patients (25 cases) with chronic non-insertional Achilles tendinopathy, equinus foot, and other indications. In the clinical study, we evaluated the range of dorsiflexion before and after the procedure, clinical outcomes with VAS and AOFAS scores, and potential complications, including neurovascular injuries.

**Results:**

We achieved complete release of the gastrocnemius tendon in all cases in the cadaveric study, with no damage to the sural nerve or vessels and minimal damage to the underlying muscle fibers. Ankle dorsiflexion increased for every patient in the study (mean, 14°; standard deviation, 3°) and was maintained throughout follow-up. The mean preoperative VAS score was 7 (6–9), which improved to 0 (0–1). The AOFAS Ankle-Hindfoot Score improved from a mean of 30 (20–40) to 93 (85–100) at 6 months. No major complications were observed. All patients returned to their previous sports after 6 months.

**Conclusions:**

After cadaveric and clinical study, we considered the technique to be safe and effective to perform ultrasound-guided ultra–minimally invasive gastrocnemius recession using a 1-mm incision in vivo. This novel technique represents an alternative to open techniques, with encouraging results and with the advantages of reducing pain, obviating lower limb ischemia, deeper anaesthesia, thus decreasing complications and contraindications and accelerating recovery.

**Electronic supplementary material:**

The online version of this article (doi:10.1186/s12891-016-1265-7) contains supplementary material, which is available to authorized users.

## Background

Isolated gastrocnemius contracture is thought to lead to numerous foot and ankle pathologic conditions. Gastrocnemius equinus is typically defined as ankle dorsiflexion <10° with the knee extended.

The resulting equinus deformity alters foot biomechanics and increases forefoot pressure, aggravating or predisposing to conditions such as Achilles tendinosis, flatfoot, lower back pain or strain, diabetic foot ulcer, knee hyperextension (genu recurvatum), metatarsalgia, plantar fasciitis, midfoot pain or arthritis, lateral foot pain, and nerve entrapment. In children, the deformity has been associated with equinus foot, spasticity, and cerebral palsy [1–12].

Therefore, gastrocnemius recession—whether alone or in combination with other techniques—has many well-documented indications. It is considered indicated in adults with dorsiflexion <10° with the knee extended [13–16].

Gastrocnemius recession can be performed as an open or an endoscopic procedure [15–19]. Many open techniques have been described, although poor cosmetic outcomes, neurovascular compromise, and potential wound complications can lead to patient dissatisfaction. Endoscopic gastrocnemius recession seems to be less invasive and may have advantages over open procedures in terms of enhanced visualization, smaller incisions, shorter operative time, fewer complications, and reduced morbidity [20]. However, both approaches still require epidural anesthesia, lower limb ischemia, and stitches.

The aim of this study was to evaluate the safety and efficacy of a new technique based on ultrasound-guided ultra–minimally invasive gastrocnemius recession. We studied the feasibility and effectiveness of the technique in cadavers and report our preliminary results in chronic non-insertional Achilles tendinopathy, equinus foot, and other indications. We evaluate the range of dorsiflexion before and after the procedure, clinical outcomes, and potential complications, including neurovascular injuries.

## Methods

We performed a pilot study with 22 cadavers to ensure that the technique was effective and safe. No neurovascular damage was observed in any of the specimens. In a second phase, we performed gastrocnemius recession in 23 patients (25 cases) with various problems. Gastrocnemius tightness was assessed clinically using the Silfverskiöld test [21]. In most patients, the procedures were combined with other ultrasound-guided ultra–minimally invasive techniques, all performed under local anesthesia plus sedation without the need for lower limb ischemia or stitches.

In 10 patients, including 1 who underwent simultaneous bilateral recession (11 cases), the indication for the procedure was non-insertional Achilles tendinopathy. In this group, ultrasound-guided Achilles tenotomy or release of the paratenon was performed with gastrocnemius recession. In 3 patients (4 cases, including the patient who underwent simultaneous bilateral recession), the indication was equinus foot. Ultrasound-guided plantar fasciotomy was performed at the same time. In 5 patients, the indication was gastrocnemius retraction in the presence of plantar fasciitis. Selective plantar fasciotomy was combined with gastrocnemius recession. In 5 patients the indication was metatarsalgia and forefoot overloading with no hammertoe or any other forefoot condition.

The study population comprised 18 males and 5 females. Mean age was 42 years (13–61). The patients with equinus foot were aged 13, 14, and 15 years. The age range of patients with Achilles tendinopathy or plantar fasciitis was 37–51 years. These included runners and triathletes. The age range of patients with metatarsalgia was 50–61 years.

All patients had previously received multiple treatments and had at least 6 months of conservative management prior to surgery, including modification of daily activity, night orthosis, stretching programs, and physical therapy. However, their symptoms failed to resolve. The average duration of symptoms ranged from 1 to 5 years.

### Surgical technique

**Fig. 1:**
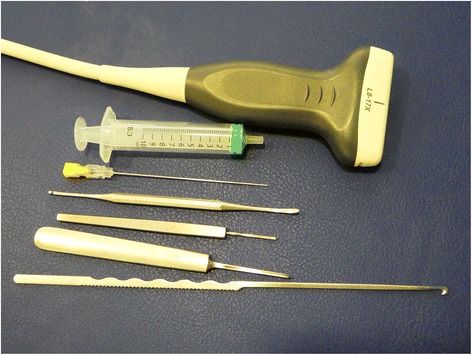
Instrument set

The instrument set included long needles (a 16-gauge, 1.7-mm diameter Abbocath; Abbott Laboratories, North Chicago, Illinois, USA), a V-shaped straight curette, a blunt dissector, a hook knife (Aesculap 2,3 mm (HH060R), and an ultrasound device (Alpinion ECube15) with a 10–17–MHz linear transducer and the Needle Vision Plus™ software package (Fig. 1).

We always perform the procedure with 2 surgeons. Thus, the first surgeon can concentrate on accurate positioning of the instruments and make the incision using 2 hands in order to steady the scalpel. The second surgeon can hold the probe and assist with technical procedures (eg, dorsal flexion of the ankle when the tendon is being severed) or insertion of the instruments.

**Fig. 2:**
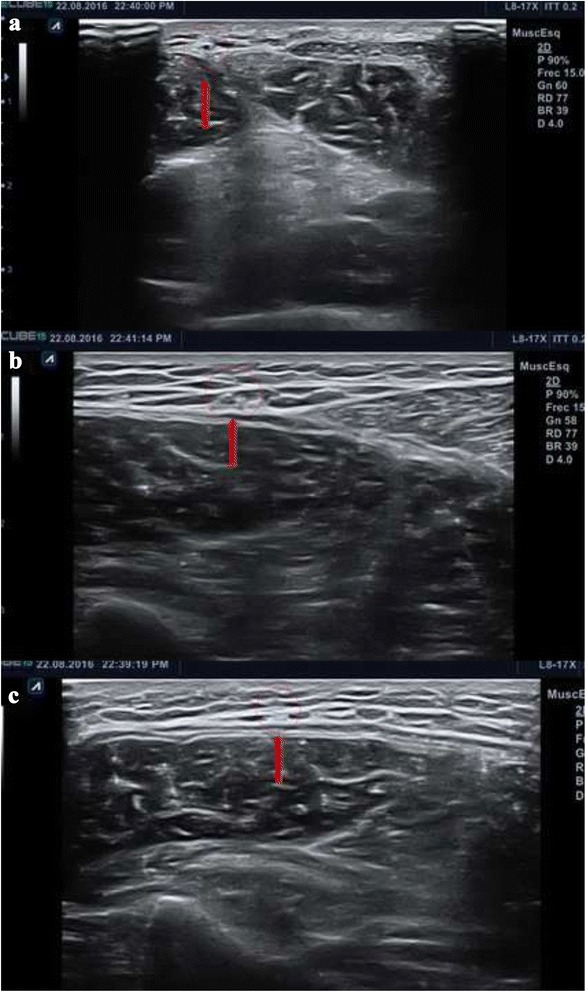
Identification of the sural nerve from distal (**a** and **b**) to proximal, where it usually crosses over the gastrocnemius tendon and becomes medial (**c**)

Ultrasound-guided ultra–minimally invasive gastrocnemius recession was performed with the patient in the prone position. No tourniquet was used, and no spinal anesthesia was required. Local anesthesia plus sedation was used when necessary. The first stage involved location of the sural nerve and vessels as this varies from person to person. The nerve is found in the distal third of the leg, near the ankle, lateral to the Achilles tendon. With the probe in the transverse plane (short axis), we follow the nerve proximally, from the distal and lateral part of the leg, at the ankle, to the proximal area, until we locate the entry point. We then mark the location of the entry point. Nerves can be identified with the probe in the transverse plane using this distal to proximal movement, since they appear as circular structures that remain constant despite the changing position of the probe (Fig. 2). On the short axis, the larger nerves are observed as a honeycomb pattern, although the sural nerve is too small for this pattern to be observed.

The nerve usually courses medially to cross over the gastrocnemius tendon in the central third of the leg.

The selected point for the recession is located 2–3 cm distal to the medial head of the gastrocnemius muscle. Recession is performed via a single 1–2–mm incision. Occasionally, 2 incisions are required to follow the shape of the gastrocnemius and complete the resection.

We prefer to place the portal close to the sural nerve in order to maintain control over the tip of the needle. At the selected point, we inject anesthesia underneath the fascia and create a working space between the nerve and the underlying fascia and the gastrocnemius tendon. This important step is easily performed with ultrasound-guided injection of anesthesia and blunt dissection. If the entry point is too far away from the nerve, it is more difficult to control the tip and direction, as the needle is flexible.

We insert 2 V-shaped straight curettes (small and medium) guided by the needle to enlarge the entry point at the fascia. Guiding the instruments towards the muscle makes it possible to cross underneath the nerve without damaging it. Sealing the entry point with betadine gel™ prevents air bubbles from entering and distorting the ultrasound image.

We then advance the blunt dissector until we reach the medial border of the gastrocnemius tendon, where we can easily insert a normal or a retractable hook knife under direct ultrasound control and advance it as far as the curve of the calf allows without perforating the superficial fascia. We insert the hook knife following the curve of the blade so as not to enlarge the incision and advance towards the medial border of the tendon in a horizontal plane and with the transducer placed in the transverse position. At the medial border of the tendon, we turn the blade 90° towards the tendon and start severing the tendon in a medial to lateral direction.

**Fig. 3:**
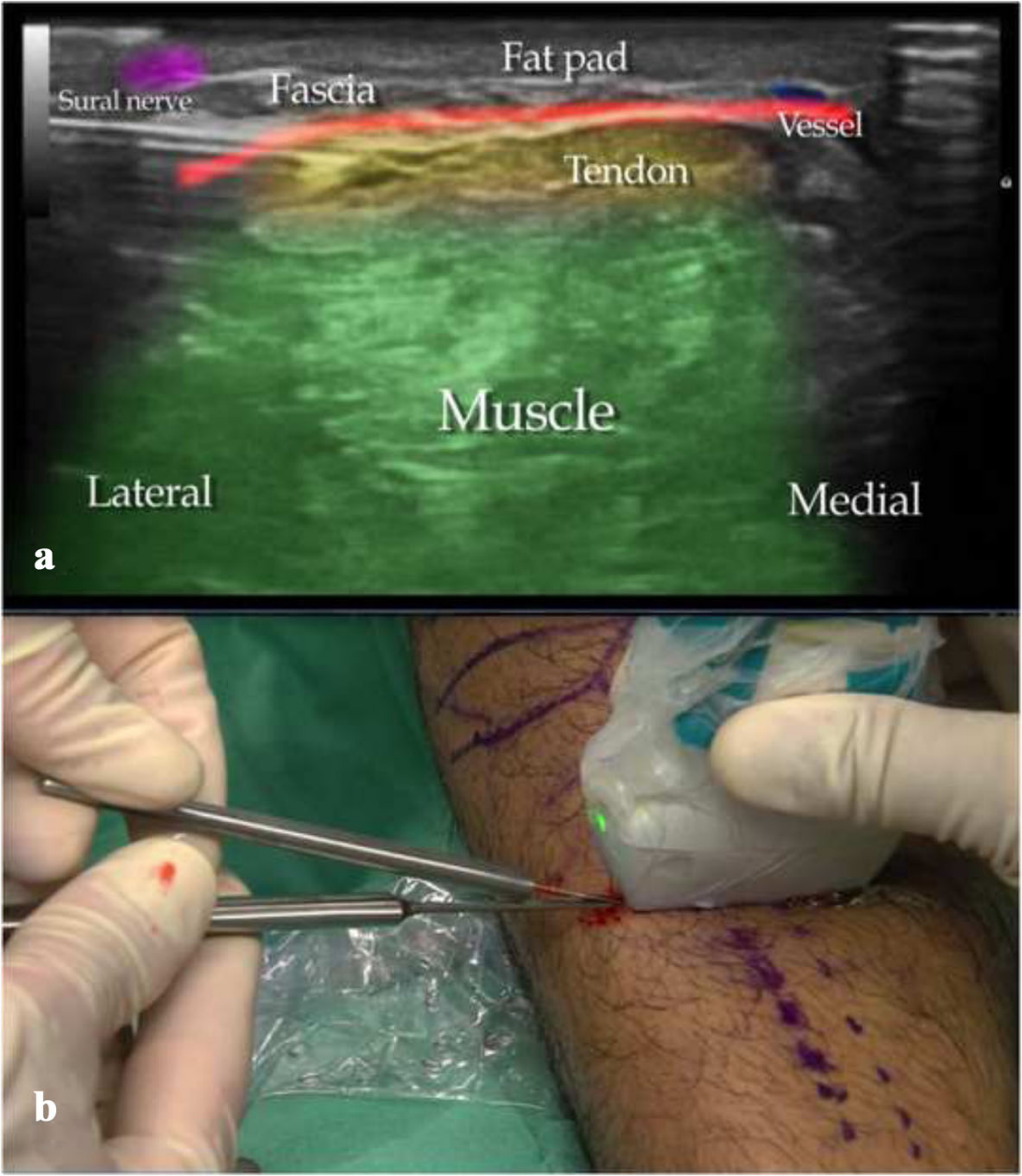
**a** Surgical technique and **b** coloured ultrasound-image

During this stage, we stretch the tendon by flexing the foot dorsally in order to maintain the position of the leg and thus avoid losing the direction of the cut. If the portal is located at the lateral border of the tendon and we can reach its medial border, we repeat this step once or twice, and the operation has ended. If the portal is more medial, we repeat the previous two steps towards the medial and lateral portion of the gastrocnemius tendon and complete the recession. If the diameter and shape of the calf make it difficult to perform the procedure in a single step, another portal is opened and the recession completed following the previous steps (Fig. 3).

We use the blunt dissector to ensure there is no tension in the tendon.

**Fig. 4:**
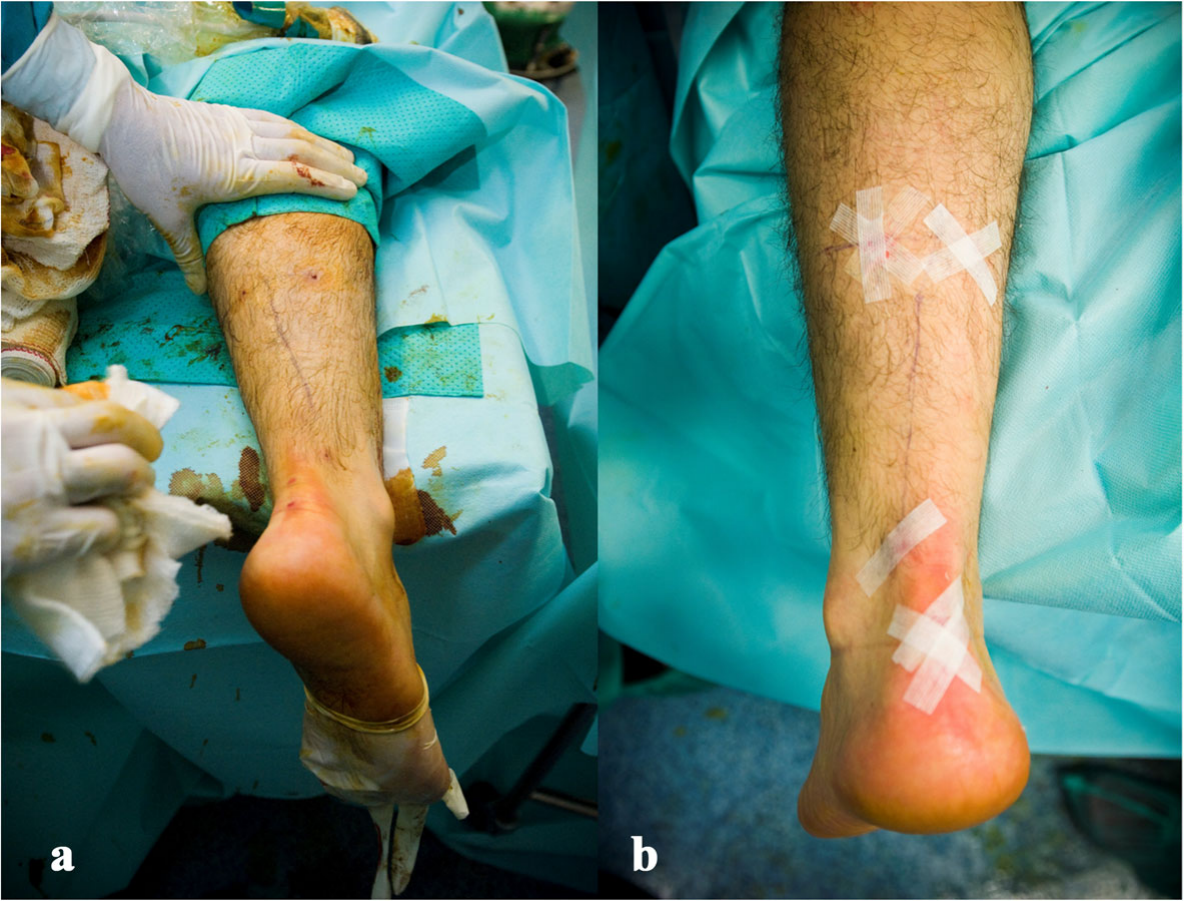
One-millimetre incisions in a combined procedure with non-insertional Achilles tendinopathy. **a** After surgery. **b** After placement of adhesive strips of the wounds

We test the range of motion of the ankle immediately after surgery, first passively. As the anesthesia is local, the patient can then actively flex the foot dorsally and plantar shortly after the procedure. No stitches are required. We use plastic adhesive strips and an elastic bandage (Fig. 4).

### Rehabilitation protocol

Active dorsiflexion and plantar flexion of the ankle are encouraged immediately after surgery. Patients are allowed to walk with elbow crutches with partial weight bearing on the day of surgery. An orthotic boot can be used in patients who have undergone Achilles tenotomy.

Full weight bearing is allowed after 3 to 7 days, depending on pain and discomfort. The boot can be removed after 1–2 weeks, although most of our patients removed it in days; therefore, we no longer recommend the orthotic boot, only the crutches. Patients can progress to a regular shoe and start physiotherapy and sports. Eccentric calf stretching exercises can be started as soon as tolerated.

### Evaluation of results

Pain was evaluated using a visual analog scale (VAS) (from 0 [no pain] to 10 [severe pain]) at baseline, 1 week, 1 month, 3 months, 6 months, and 1 year after surgery in all but 1 patient, whose follow-up was shorter. Pain was also evaluated using the Ankle-Hindfoot Score of the American Orthopaedic Foot and Ankle Society (AOFAS) (pain, 40 points; function, 50 points; alignment, 10 points) at baseline, 1 month, 3 months, 6 months, and 1 year after surgery.

Other variables, such as the ability to support autonomous comfortable plantigrade weight bearing after surgery, calf strength, or days on painkillers were also analyzed. Complications were recorded.

We assessed statistical differences between preoperative and postoperative functional outcome (VAS and AOFAS Ankle-Hindfoot Score) using repeated measures non parametric Friedman test, for more than two measures, and Wilcoxon Signed-rank test to identify where the specific differences lie using the Bonferroni correction.

Statistical analysis was performed using SPSS 11.0 for Windows, and statistical significance was set at α < 0.05 using a 2-tailed test.

## Results

### Cadaver study

We achieved complete release of the gastrocnemius tendon in all the cadavers, with no damage to the sural nerve or vessels. Minimal damage to the underlying muscle fibers was observed. We considered the technique to be sufficiently safe and effective to perform ultrasound-guided gastrocnemius recession using a 1-mm incision in vivo.

### Clinical study

In the clinical series, ankle dorsiflexion increased for every patient in the study (mean, 14°; standard deviation, 3°). Postoperative dorsiflexion was maintained for all patients throughout follow-up. There were no statistically significant differences in ankle dorsiflexion measurements between isolated and combined procedures at any time point (Additional files 1 and 2).

**Fig. 5:**
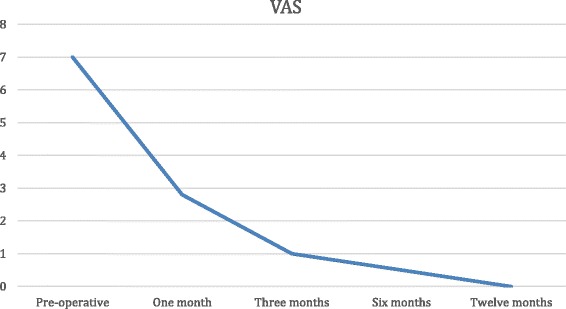
Progress of VAS over time

**Fig. 6:**
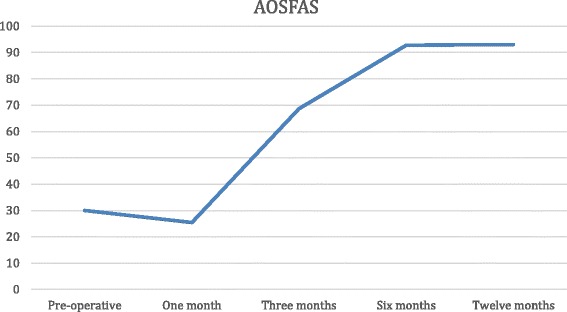
Progress of AOFAS over time

Pain and function improved significantly in all patients. The mean preoperative VAS score was 7 (6–9), which improved to 2.8 at 3 months, 1 at 6 months and 0 (0–1) at the most recent follow-up visit, at 12 months. The AOFAS Ankle-Hindfoot Score improved from a mean of 30 (20–40) to 25.42 (20–35) at 1 month, 68.76 (44–82) at 3 months, 92.84 (85–100) at 6 months, and 93 at 1 year. Therefore, improvement in pain was significant at 1 month, although improvement in function was significant and stabilized at 6 months (Figs. 5 and 6).

All runners, triathletes, and occasional athletes returned to their previous sports and running after 6 months. One patient experienced occasional discomfort that made it difficult for him to run long distances.

### Complications

All patients developed mild superficial hematomas that resolved in 3–4 weeks. These were worse in patients with non-insertional Achilles tendinopathy who had to undergo several surgical procedures simultaneously. During rehabilitation, some patients developed minor internal late hematomas or seromas (not visible on clinical observation, observed by ultrasound) at the areas of the tendon and muscle surrounding the recession until the third month. Four patients experienced slight weakness, which resolved at 6 months. There were no instances of over-lengthening or Achilles tendon rupture.

We observed no infections, wound complications, or nerve complications (eg, nerve damage, numbness). The scars were not visible in most patients at the most recent follow-up visit.

## Discussion

The many indications for gastrocnemius recession in the literature includenon-insertional Achilles tendinopathy, flatfoot, lower back pain or strain, knee hyperextension (genu recurvatum), plantar fasciitis, midfoot pain or arthritis, lateral foot pain, and nerve entrapment. It is also indicated in children with equinus foot deformity, spasticity, or cerebral palsy [3–9].

Gastrocnemius recession can be performed as an open or an endoscopic procedure. The many open techniques described [15–19] include distal gastrocnemius recession (Strayer), proximal gastrocnemius release from the femoral condyles (Silfverskiöld), division of the gastrocnemius aponeurosis (Vulpius), and various open and percutaneous Achilles tendon lengthening procedures. Patient dissatisfaction with open procedures is generally due to poor cosmesis, neurovascular compromise, and potential wound complications. Endoscopic gastrocnemius recession is less invasive and may have advantages over open procedures in terms of enhanced visualization, smaller incisions, shorter operative time, fewer complications, and less morbidity [20]. However, both open and endoscopic recessions still require epidural anesthesia, lower limb ischemia, and stitches. In addition, there is a real possibility of damaging the sural nerve, which is not under the complete control of the surgeon at all stages of the procedure.

Ultra–minimally invasive surgery has been defined as surgery that requires a 1-mm incision such as that left by a 16-gauge (1.7-mm-diameter) Abbocath (Abbott Laboratories, North Chicago, Illinois, USA) [22]. To our knowledge, ultrasound-guided ultra–minimally invasive gastrocnemius recession has not been described elsewhere, and many of the cases described herein are novel in the literature. The procedure proved to be safe and effective in both the pilot cadaver study and in the clinical series. Clinical outcomes were significantly better than before surgery. The range of dorsiflexion improved without weakness in the calf muscles, and there were no relevant complications. Hematomas developed in most cases.

Ultrasound-guided procedures allow continuous visualization of nerves and vessels, unlike endoscopic procedures, where the nerve has to be identified after inserting the instruments and camera, thus placing it at risk during the initial stages. The lack of numbness or paresthesias in our series is significant and encouraging.

In the present series, pain was minimal. Some patients took medication for only 1 to 3 days.

This novel procedure may improve on all the claimed advantages of endoscopic techniques over open techniques. We strongly believe that an ultra–minimally invasive procedure is not performed merely to reduce the size of the incision and ensure better cosmesis. It has more to do with preventing pain, obviating lower limb ischemia, and, therefore, deeper anesthesia. Thus we can reduce complications and contraindications and accelerate recovery.

All the procedures were performed under local anesthesia plus sedation in an outpatient regimen, without lower limb ischemia. No sutures were necessary. A key advantage of our approach is the possibility of combining ultrasound-guided ultra–minimally invasive techniques to ensure minimal pain with excellent outcomes and no significant morbidity.

Gastrocnemius recession is one of several surgical procedures for non-insertional Achilles tendinopathy, plantar fasciitis, and associated problems; however, the best option remains controversial [11, 12, 23–30]. All these procedures can be performed under ultrasound guidance in an ultra–minimally invasive way [31, 32].

Although our preliminary results are encouraging, large randomized controlled trials are necessary to evaluate available techniques and to establish the real advantages of combining surgical techniques in many lower limb disorders.

## Conclusions

Ultrasound-guided, ultra–minimally invasive surgery is an emerging technology that gives the surgeon direct control over the main structures and may be the future gold-standard for many surgical procedures such as plantar fasciotomy, gastrocnemius recession, and treatment of non-insertional Achilles tendinopathy. However, the learning curve is steep, because the surgeon has to perfect the technique with cadavers and become competent in the use of ultrasound.

Two videos illustrating these surgical techniques are included in the Educational Media Program of the American Academy of Orthopaedic Surgeons (AAOS) and are available upon request [31, 32].

## Additional files

Additional file 1: Descriptive and statistical data: Descriptive data from the VAS and AOFAS and results of the Friedman and Wilcoxon tests. (DOCX 43 kb) Additional file 2: Clinical and functional data: Range of motion, AOFAS, VAS, and complications after gastrocnemius lengthening. (XLSX 10 kb)

## Abbreviations

AAOS: American Academy of Orthopaedic Surgeons
AOFAS: American Orthopaedic Foot and Ankle Society score
MHz: Megahertz
VAS: Visual analog scale
