# Supplementary material for: Ultrasound-guided gastrocnemius recession: a new ultra–minimally invasive surgical technique
Source: BMC Musculoskelet Disord. 2016 Oct 3;17:409. doi: 10.1186/s12891-016-1265-7 (PMC5048654; doi:10.1186/s12891-016-1265-7)
Supplement: Additional file 1: — Descriptive and statistical data: Descriptive data from the VAS and AOFAS and results of the Friedman and Wilcoxon tests. (DOCX 43 kb) [file 12891_2016_1265_MOESM1_ESM.docx]

| **Descriptive statistics** | | | | | | |
| --- | --- | --- | --- | --- | --- | --- |
|  | N | Rank | Minimum | Maximum | Mean | Standard deviation |
| VAS_PREOP | 25 | 3,00 | 6,00 | 9,00 | 6,9600 | ,88882 |
| VAS_1_MONTH | 25 | 4,00 | 1,00 | 5,00 | 2,8000 | 1,11803 |
| VAS_3_MONTHS | 25 | 3,00 | ,00 | 3,00 | ,9600 | ,88882 |
| VAS_1_YEAR | 25 | 1,00 | ,00 | 1,00 | ,4000 | ,50000 |
| AOSFAS_PREOP | 25 | 20,00 | 20,00 | 40,00 | 30,4400 | 5,93773 |
| AOSFAS_1_MONTHWEEK | 25 | 15,00 | 20,00 | 35,00 | 25,9200 | 4,45271 |
| AOSFAS_3_MONTHS | 25 | 38,00 | 44,00 | 82,00 | 68,7600 | 8,85193 |
| AOSFAS_6_MONTHS | 25 | 15,00 | 85,00 | 100,00 | 92,8400 | 4,91325 |
| N válido (según lista) | 25 |  |  |  |  |  |

DESCRIPTIVE VARIABLES VAS Y ASOSFAS

**VAS study**

**Non parametric test**

| **Descriptive statistics** | | | | | | | | |
| --- | --- | --- | --- | --- | --- | --- | --- | --- |
|  | N | Media | Standard deviation | Minimum | Maximum | Percentiles | | |
|  |  |  |  |  |  | 25 | 50 (median) | 75 |
| VAS_PREOP | 25 | 6,9600 | ,88882 | 6,00 | 9,00 | 6,0000 | 7,0000 | 8,0000 |
| VAS_1_MONTH | 25 | 2,8000 | 1,11803 | 1,00 | 5,00 | 2,0000 | 3,0000 | 4,0000 |
| VAS_3_MONTHS | 25 | ,9600 | ,88882 | ,00 | 3,00 | ,0000 | 1,0000 | 1,0000 |
| VAS_1_YEAR | 25 | ,4000 | ,50000 | ,00 | 1,00 | ,0000 | ,0000 | 1,0000 |

**Friedman test**

| **Ranks pain analysis** | |
| --- | --- |
|  | Mean rank |
| VAS_PREOP | 4,00 |
| VAS_1_MONTH | 3,00 |
| VAS_3_MONTHS | 1,64 |
| VAS_1_YEAR | 1,36 |

| **Test statistics ^a^** | |
| --- | --- |
| N | 25 |
| Chi--square | 73,371 |
| gl | 3 |
| Sig. asintót. | ,000 |
| a. Friedman’s test | |

THIS P VALOR MEANS THAT THE EVOLUTION WAS SIGNIFICATIVE IN THE FOLLOW-UP: Friedman’s test

To compare in pairs, we used non parametric Wilcoxon Signed-rank test with the Bonferroni correction (α = .05 / number of comparisons) to interpret the results.

We have 4 measurements. The correct p value to use was 0.0125 (0.05/4 = 0.0125)

**Non-parametric test**

| **Descriptive statistics** | | | | | |
| --- | --- | --- | --- | --- | --- |
|  | N | Mean | Standard deviation | Minimum | Maximum |
| VAS_PREOP | 25 | 6,9600 | ,88882 | 6,00 | 9,00 |
| VAS_1_MONTH | 25 | 2,8000 | 1,11803 | 1,00 | 5,00 |
| VAS_3_MONTHS | 25 | ,9600 | ,88882 | ,00 | 3,00 |
| VAS_1_YEAR | 25 | ,4000 | ,50000 | ,00 | 1,00 |

**Wilcoxon Signed Rank test**

| **Ranks** | | | | |
| --- | --- | --- | --- | --- |
|  |  | N | Average range | Rank sum |
| VAS_1_MONTH - VAS_PREOP | Negative ranks | 25^a^ | 13,00 | 325,00 |
|  | Positive ranks | 0^b^ | ,00 | ,00 |
|  | Draw ranks | 0^c^ |  |  |
|  | Total | 25 |  |  |
| VAS_3_MONTHS - VAS_PREOP | Negative ranks | 25^d^ | 13,00 | 325,00 |
|  | Positive ranks | 0^e^ | ,00 | ,00 |
|  | Draw ranks | 0^f^ |  |  |
|  | Total | 25 |  |  |
| VAS_1_YEAR - VAS_PREOP | Negative ranks | 25^g^ | 13,00 | 325,00 |
|  | Positive ranks | 0^h^ | ,00 | ,00 |
|  | Draw ranks | 0^i^ |  |  |
|  | Total | 25 |  |  |
| a. VAS_1_MONTH < VAS_PREOP | | | | |
| b. VAS_1_MONTH > VAS_PREOP | | | | |
| c. VAS_1_MONTH = VAS_PREOP | | | | |
| d. VAS_3_MONTHS < VAS_PREOP | | | | |
| e. VAS_3_MONTHS > VAS_PREOP | | | | |
| f. VAS_3_MONTHS = VAS_PREOP | | | | |
| g. VAS_1_YEAR < VAS_PREOP | | | | |
| h. VAS_1_YEAR > VAS_PREOP | | | | |
| i. VAS_1_YEAR = VAS_PREOP | | | | |

| **Test statistics ^b^** | | | |
| --- | --- | --- | --- |
|  | VAS_1_MONTH - VAS_PREOP | VAS_3_MONTHS - VAS_PREOP | VAS_1_YEAR - VAS_PREOP |
| Z | -4,425^a^ | -4,446^a^ | -4,442^a^ |
| Sig. (bilateral) | ,000 | ,000 | ,000 |
| a. Ranks positive based. | | | |
| b. Wilcoxon Signed Rank test | | | |

This last table shows every change compared with preoperative situation were significant with p<0.0125

**AOSFAS analysis**

**Non-parametric test**

| **Descriptive statistics** | | | | | | | | |
| --- | --- | --- | --- | --- | --- | --- | --- | --- |
|  | N | Mean | Standard deviation | Minimum | Maximum | Percentiles | | |
|  |  |  |  |  |  | 25 | 50 (median) | 75 |
| AOSFAS_PREOP | 25 | 30,4400 | 5,93773 | 20,00 | 40,00 | 26,0000 | 32,0000 | 35,0000 |
| AOSFAS_1_MONTHWEEK | 25 | 25,9200 | 4,45271 | 20,00 | 35,00 | 22,0000 | 26,0000 | 29,0000 |
| AOSFAS_3_MONTHS | 25 | 68,7600 | 8,85193 | 44,00 | 82,00 | 64,5000 | 70,0000 | 75,0000 |
| AOSFAS_6_MONTHS | 25 | 92,8400 | 4,91325 | 85,00 | 100,00 | 90,0000 | 93,0000 | 97,0000 |

**Friedman´s test**

| **Ranks** | |
| --- | --- |
|  | Average range |
| AOSFAS_PREOP | 1,90 |
| AOSFAS_1_MONTHWEEK | 1,10 |
| AOSFAS_3_MONTHS | 3,00 |
| AOSFAS_6_MONTHS | 4,00 |

| **Test statistics ^a^** | |
| --- | --- |
| N | 25 |
| Chi-square | 73,178 |
| gl | 3 |
| Sig. | ,000 |
| a. Friedman´s test | |

As VAS analysis, the evolution is significant

**Non-parametric test**

| **Descriptive statistics** | | | | | |
| --- | --- | --- | --- | --- | --- |
|  | N | Mean | Standard deviation | Minimum | Maximum |
| AOSFAS_PREOP | 25 | 30,4400 | 5,93773 | 20,00 | 40,00 |
| AOSFAS_1_MONTHWEEK | 25 | 25,9200 | 4,45271 | 20,00 | 35,00 |
| AOSFAS_3_MONTHS | 25 | 68,7600 | 8,85193 | 44,00 | 82,00 |
| AOSFAS_6_MONTHS | 25 | 92,8400 | 4,91325 | 85,00 | 100,00 |

**Wilcoxon Signed Rank test**

| **Ranks** | | | | |
| --- | --- | --- | --- | --- |
|  |  | N | Average range | Rank sum |
| AOSFAS_1_MONTHWEEK - AOSFAS_PREOP | Negative ranks | 21^a^ | 11,93 | 250,50 |
|  | Positive ranks | 1^b^ | 2,50 | 2,50 |
|  | Draw ranks | 3^c^ |  |  |
|  | Total | 25 |  |  |
| AOSFAS_3_MONTHS - AOSFAS_PREOP | Negative ranks | 0^d^ | ,00 | ,00 |
|  | Positive ranks | 25^e^ | 13,00 | 325,00 |
|  | Draw ranks | 0^f^ |  |  |
|  | Total | 25 |  |  |
| AOSFAS_6_MONTHS - AOSFAS_PREOP | Negative ranks | 0^g^ | ,00 | ,00 |
|  | Positive ranks | 25^h^ | 13,00 | 325,00 |
|  | Draw ranks | 0^i^ |  |  |
|  | Total | 25 |  |  |
| a. AOSFAS_1_MONTHWEEK < AOSFAS_PREOP | | | | |
| b. AOSFAS_1_MONTHWEEK > AOSFAS_PREOP | | | | |
| c. AOSFAS_1_MONTHWEEK = AOSFAS_PREOP | | | | |
| d. AOSFAS_3_MONTHS < AOSFAS_PREOP | | | | |
| e. AOSFAS_3_MONTHS > AOSFAS_PREOP | | | | |
| f. AOSFAS_3_MONTHS = AOSFAS_PREOP | | | | |
| g. AOSFAS_6_MONTHS < AOSFAS_PREOP | | | | |
| h. AOSFAS_6_MONTHS > AOSFAS_PREOP | | | | |
| i. AOSFAS_6_MONTHS = AOSFAS_PREOP | | | | |

| **Test statistics ^c^** | | | |
| --- | --- | --- | --- |
|  | AOSFAS_1_MONTHWEEK - AOSFAS_PREOP | AOSFAS_3_MONTHS - AOSFAS_PREOP | AOSFAS_6_MONTHS - AOSFAS_PREOP |
| Z | -4,045^a^ | -4,375^b^ | -4,376^b^ |
| Sig.. (bilateral) | ,000 | ,000 | ,000 |
| a. Positive ranks based. | | | |
| b. Negative ranks based. | | | |
| c. Wilcoxon Signed Rank test | | | |

The comparisons between all measurements from preoperative values were significant with p p<0.0125
